# Fasting in critical illness: the role of ketonuria — a retrospective observational study

**DOI:** 10.1186/s44158-024-00199-7

**Published:** 2024-09-16

**Authors:** Irene Ottaviani, Simona Tantillo, Lorenzo Miggiano, Martina Guarnera, Marco Menghini, Francesco Talarico, Federica Mazzanti, Nicola Cilloni

**Affiliations:** Department of Anesthesia, Intensive Care and Prehospital Emergency, Maggiore Hospital Carlo Alberto Pizzardi, Bologna, Italy

**Keywords:** Ketonuria, Metabolic acidosis, Perioperative fasting, Catabolic pathways, Anabolic resistance

## Abstract

**Background:**

Metabolic acidosis is a frequent finding in patients admitted to the intensive care unit (ICU). It can be caused by prolonged fasting due to surgical procedures or by medical conditions that lead to starvation ketoacidosis (SKA). Early recognition and treatment of SKA could prevent several life-threatening complications, improving survival and reducing the ICU length of stay.

**Methods:**

We retrospectively screened all medical records of patients admitted to the ICU (Maggiore Hospital, Bologna, North Italy) from May 2022 to April 2023. We included patients aged 18 years or older who presented ketonuria detected in the urine sample.

**Results:**

We analyzed 190 patients with ketonuria at ICU admission. Postsurgical patients showed lower levels of albumin and a higher rate of shock compared to medical patients. Ketonuric patients with shock had a lower body mass index (BMI) compared to patients without shock (24 versus 26 kg/m2, respectively). There were no differences within groups regarding mortality and ICU readmission rate. Medical patients had a significantly higher ICU length of stay.

**Conclusions:**

This retrospective observational descriptive study showed that patients with ketonuria, hypoalbuminemia, and low BMI at ICU admission have high risk of hemodynamic instability and shock. Surgical patients compared to medical patients are exposed to a catabolic trigger that could worsen a state of malnutrition and induce anabolic resistance; elective and urgent surgical patients did not differ in terms of risk of shock and mortality, probably due to the activation of this catabolic pathway. Early recognition and treatment of starvation ketoacidosis and perioperative nutritional optimization could reduce incidence of hemodynamic and metabolic complications.

## Introduction

**Fig. 1:**
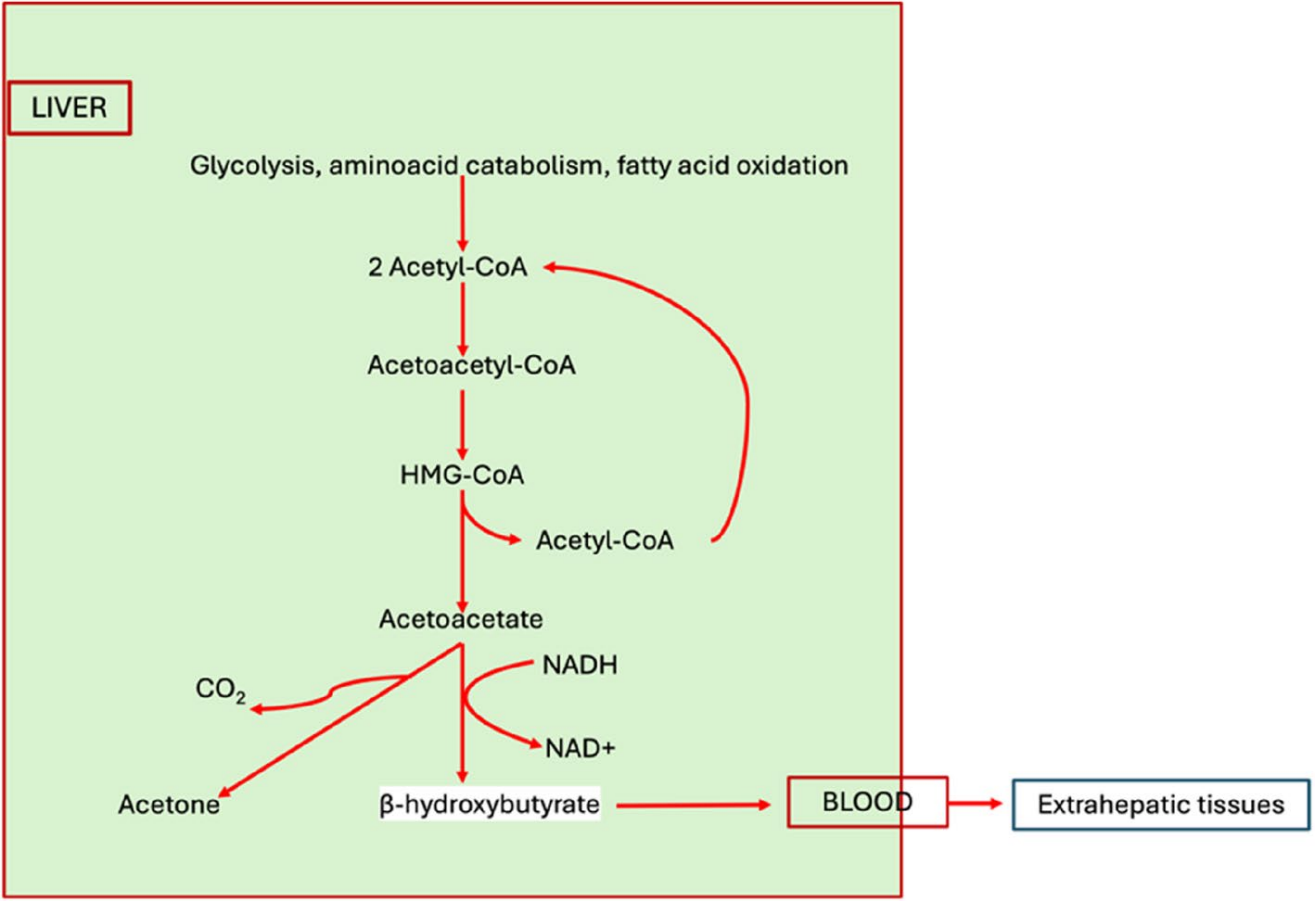
Ketone bodies production

Metabolic acidosis is a frequent finding in patients admitted to the intensive care unit (ICU); one of the cause could be starvation ketoacidosis (SKA) that can be caused by prolonged fasting [1, 2]. Prolonged fasting and consequent glucose deficiency activate glycogenolysis and gluconeogenesis and inhibit glycolysis. The Krebs cycle is inhibited due to lack of oxaloacetate which is oxidized to phosphoenolpyruvate for gluconeogenesis, and the acetyl-CoA deriving from the oxidation of fatty acids is used for the synthesis of ketone bodies: acetoacetate, *β*-hydroxybutyrate, and acetone [3, 4] (Fig. 1).

In healthy adults, the glycogen is stored in skeletal muscle, almost 80%, and in the liver, where glycogen particles contain the highest amount of glucose molecules. Other organs, like the heart and brain, contain lower glycogen stores. Glycogen stores are depleted after 24 h of fasting, and the body must use alternative energy stores to maintain its metabolism. Glucagon stimulates the lipases and determines further activation of lipolysis and acid fat chains transport to liver mitochondria for beta oxidation [5]. In case of high-energy requirements, leucine and isoleucine can be used as substrates for acetyl-CoA production and ketogenesis [6, 7]. In healthy adults, ketone bodies can be used as energy substrates: after being produced by liver mitochondria, they enter extrahepatic tissues and go through reoxidation to produce acetyl-CoA that is used in the Krebs cycle. The use of ketone bodies to produce energy limits the catabolism of amino acids [7]. In normal conditions, an increase in blood ketone concentrations inhibits further activation of lipolysis, increases insulin production, and inhibits further formation of ketone bodies [8]. The excessive production of ketone bodies can lead to a condition of metabolic acidosis (i.e., ketoacidosis) that is an alteration of the acid-base balance in which there is a reduction of bicarbonate concentration in the blood and a low pH [9]. In ICU patients, metabolic acidosis is associated with an increased mortality risk, due to sepsis, arrhythmic events, myocardial dysfunction, cerebral edema, pulmonary vasoconstriction, systemic vasodilation, and longer length of stay [10, 11].

Other frequent causes of acidosis are septic shock, acute renal failure, drug intoxication, hypovolemia/hemorrhagic shock, and cardiac arrest [9, 12]. Starvation ketoacidosis (SKA) needs to be differentiated from alcoholic ketoacidosis (AKA) and diabetic ketoacidosis (DKA) [13–15]. Sometimes, these causes of acidosis coexist, and it is not possible to make a differential diagnosis [13–15].

SKA can cause metabolic acidosis, which does not respond to volume expansion and administration of bicarbonates, and hemodynamic instability, which requires vasopressor support and hospitalization in an intensive care environment. Patients in the perioperative period [16, 17], those suffering from pancreatitis [18, 19], and septic patients [20] appear to be more exposed to this risk, probably due to stress-induced hormonal dysregulation (increased cortisol levels, reduced insulin/glucagon ratio).

In critically ill patients, there are several factors that can contribute to the development of ketone bodies production and SKA [21–24]. Fasting, associated with the stress response to surgery and to critical illness, could lead to the development of a catabolic state and anabolic dysregulation [2].

The resolution of this clinical condition is often rapid after glucose administration or refeeding. In the absence of specific treatment guidelines, indications for treatment of diabetic ketoacidosis are usually used, e.g., infusion of glucosalin 10% 125 ml/h (https://www.bsped.org.uk/media/1798/bsped-dka-guideline-2020.pdf). In these patients, it is also necessary to keep in mind the risk of developing the refeeding syndrome [25]. SKA is considered a relatively rare condition.

Ketoacidosis in emergency department and ICU patients is reported just in a few case reports and case series [26]. It is not known why some critically ill patients use ketones early to produce energy and which patients are more at risk of developing ketoacidosis. The aim of this study is to evaluate the incidence of ketonuria in patients admitted to the ICU and to analyze hemodynamic or metabolic complications associated with it.

## Materials and methods

### Inclusion and exclusion criteria

Patients were included in the study if aged 18 years or older and with positive ketonuria at ICU admission or within the first day of the ICU stay. Exclusion criteria were the following: severe chronic kidney disease (CKD) or acute kidney injury (AKI) KDIGO III/IV/V [27], severe chronic obstructive pulmonary disease (COPD group C/D and E) [28], diabetes, high levels of glycosuria and hyperglycemia > 270 mg/dl at ICU admission [29], septic shock, and drugs intoxication [15].

### Data collection

Data related to demographic characteristics (age, sex, BMI), comorbidity (Charlson Comorbidity Index, CCI [30], SAPS II [31], amine support requirement, laboratory data, and ICU and hospital outcomes were collected from digital records.

### Outcome

The outcome of the study was to evaluate the incidence of ketonuria in patients admitted to the ICU and its role in the development of metabolic dysregulation and hemodynamic instability.

### Statistical analysis

Data are presented as mean and standard deviation for continuous variables with normal distribution, as median and interquartile range for not normally distributed data or for subgroups of the population with different sample sizes and as percentage for categorical variables. For continuous variables with normal distribution, the Student’s *T*-test was used for the comparison between two groups and the one-way ANOVA for the comparison between three groups followed by the Bonferroni post hoc test for the comparison between subgroups. For categorical variables, the chi-square test was used, and for the comparison of non-normally distributed variables or for subgroups with different simple sizes, the nonparametric Mann-Whitney test or the Kruskal-Wallis test for independent variables was used. Statistical significance is given by *p*-value < 0.05.

## Results

**Fig. 2:**
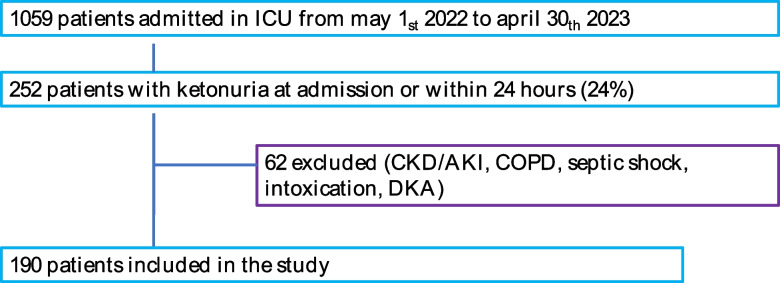
Study flowchart

From May 2022 to April 2023, 1059 patients were admitted to the ICU, and 252 (24%) of these had ketonuria at admission or within the first 24 h of ICU stay: 62 patients were excluded because they met the exclusion criteria, and 190 patients were included in the study (Fig. 2). The fasting time of the study population was more than 12 h.

Admission to the ICU was due to medical reasons in 62 patients (32%), due to respiratory failure for pneumonia (*n* = 24), pneumothorax (*n* = 3), or pulmonary edema (*n* = 5), sepsis (*n* = 8), acute neurological impairment (*n* = 11), trauma (*n* = 7), cardiogenic shock (*n* = 2), pancreatitis (*n* = 1), and cardiac arrest (*n* = 1). It followed urgent/emergency surgery in 68 (35.7%) patients, after major gastrointestinal surgery (*n* = 40), thoracic surgery (*n* = 6), urologic surgery (*n* = 5), vascular surgery (*n* = 10), and trauma (*n* = 7), and it followed elective surgery in 60 patients (31.5%), after major gastrointestinal surgery (*n* = 28), thoracic surgery (*n* = 7), urologic surgery (*n* = 7), vascular surgery (*n* = 2), orthopedic surgery (*n* = 5), vertebral surgery (*n* = 2), trauma (*n* = 2), gynecological surgery (*n* = 2), thyroidectomy (*n* = 1), and minor surgery (*n* = 4). The mean age of the population was 63 ± 15 years, with a statistically significant higher value in the elective surgical population (68 ± 12, *p* = 0.016); the CCI was significantly higher in this subgroup (5.3 ± 3 versus 4.2 ± 2.9 in medical patients and 3.9 ± 3 in urgent surgical patients, *p* = 0.01). Within groups, there were no significant differences regarding sex and BMI (Table 1).

**Table 1:** Demographic variables of the study population

	**Tot. *n* = 190**	**Medical *n* = 62**	**E surgery *n* = 60**	**U surgery *n* = 68**	***p*-value**
Age	63 ± 15	62 ± 17	68 ± 12	60 ± 16	0.016
BMI kg/m^2^	26.1 ± 5.3	26.2 ± 4.7	25.6 ± 4.6	26.4 ± 6.3	0.67
Sex					
F	*n* = 80	*n* = 20 (32%)	*n* = 28 (47%)	*n* = 32 (47%)	0.16
M	*n* = 110	*n* = 42 (67%)	*n* = 32 (53%)	*n* = 36 (53%)	
CCI	4.5 ± 3	4.2 ± 2.9	5.3 ± 3	3.9 ± 3	0.01

*E surgery* Elective surgery, *U surgery* Urgent surgery

Comparing the different subgroups of ketonuric patients, elective surgery patients had a lower SAPS II at admission compared to medical and urgent surgery patients (34 ± 14 versus 42 ± 17 in medical patients and 42 ± 21 in urgent surgical patients, *p* = 0.022), significantly higher albumin levels compared to urgent surgical patients (27 ± 4.3 g/L versus 25 ± 5.6 g/L, *p* < 0.001) but significantly lower if compared to medical patients (27 ± 4.3 g/L versus 29 ± 5.6 g/L, *p* < 0.001); elective surgical patients also have lower levels of blood bicarbonates (23.35 ± 2.9 mmol/L versus 24.05 ± 5.2 mmol/L of urgent surgical patients and 25.72 ± 5.42 mmol/L of medical patients, *p* = 0.022). Mean mNUTRIC score was 3.5 ± 2, and it did not differ significantly between subgroups.

**Fig. 3:**
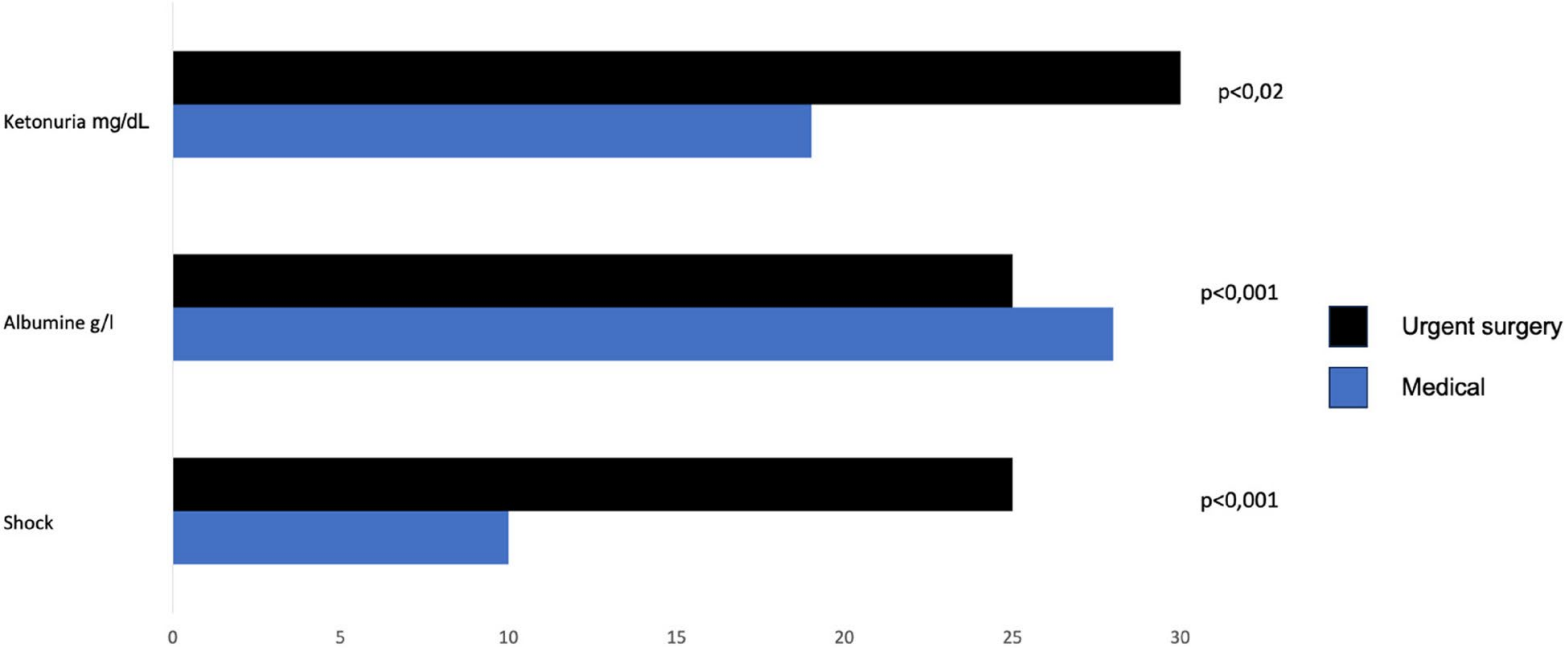
Medical versus urgent surgical patients: ketonuria, albumin, and shock

Surgical patients with ketonuria presented a greater rate of shock at ICU admission compared to medical patients (elective surgery 26.7%, urgent surgery 38.2%, medical patients 14.5%, *p* = 0.01) (Table 2, Fig. 3).

**Table 2:** Variables at ICU admission and different subgroups of ketonuric patients

	**Tot. *n* = 190**	**Medical *n* = 62**	**E surgery *n* = 60**	**U surgery *n* = 68**	***p*-value**
SAPS II	40 ± 18	42 ± 17	34 ± 13	42 ± 21	0.022
mNutric	3.5 ± 2	3.6 ± 2	3.15 ± 1.3	3.2 ± 2.2	0.7
BMI kg/m^2^	26.1 ± 5.3	26.2 ± 4.7	25.6 ± 4.6	26.4 ± 6.3	0.67
Albumin g/L	27 ± 5.5	29 ± 5.6	27 ± 4.3	25 ± 5.6	< 0.001
Ketonuria mg/dL	24.76 ± 26.8	19.5 ± 20.4	24.9 ± 24.6	29.4 ± 32.6	0.129^*^
HCO3 − mmol/L	24.38 ± 4.78	25.72 ± 5.42	23.35 ± 2.9	24.05 ± 5.2	0.022
pH	7.42 ± 0.3	7.48 ± 0.52	7.39 ± 0.56	7.39 ± 0.74	0.21
BE mmol/L	− 0.5 ± 6.9	0.28 ± 9.4	− 0.91 ± 4.6	− 0.93 ± 6	0.55
Glycemia mg/dL	137 ± 41	134 ± 44	151 ± 41	129 ± 37.9	0.002
Hemoglobin g/dL	10.93 ± 2	11 ± 2	10.9 ± 2	10.88 ± 2	0.06
LAC mmol/L	1.37 ± 1.79	1.57 ± 2.55	1.3 ± 0.84	1.25 ± 1.57	0.57
Shock	*n* = 51 (26.8%)	*n* = 9 (14.5%)	*n* = 16 (26.7%)	*n* = 26 (38.2%)	0.01

*E surgery* Elective surgery, *U surgery* Urgent surgery

^*^One-way ANOVA, post hoc Bonferroni test statistically significant medical patients compared to U surgery, *p* = 0.004

At ICU admission, 51 patients presented a state of shock (mean arterial pressure < 65 mmHg despite fluid load, need for amine support): 9 in the medical group (17%), 16 elective surgery patients (32%), and 26 patients admitted after urgent surgery (51%) (Pearson chi-square *p* = 0.01) (Table 2). The patients with shock had significantly lower levels of serum albumin and blood bicarbonates compared to patients without shock (22.6 (20–25) g/L and 22 (20–25) mmol/L versus 28.3 (25–32) and 25 (23–27) mmol/L, respectively, *p* = 0.015). Ketonuric patients with shock had lower BMI (24 kg/m^2^ versus 26 kg/m^2^, *p* = 0.02) and a higher ICU mortality compared to patients without shock (15% versus 5.7%, *p* = 0.03) (Table 3).

**Table 3:** Ketonuric patients: shock versus no shock

	**Shock *n* = 51**	**No shock *n* = 139**	***p*-value**
BMI kg/m^2^	24 (22–27)	26 (23–29)	0.02
Albumin g/L	22.6 (20–25)	28.3 (25–32)	0.009
HCO3 − mmol/L	22 (20–25)	25 (23–27)	0,015
BE mmol/L	− 2.25 (− 6.1–0.6)	0.65 (− 2–3)	0,23
ICU mortality	*n* = 8 (15%)	*n* = 8 (5.7%)	0,03

There are no differences within groups regarding mortality and ICU readmission rates. Patients admitted after surgery, both urgent and elective, showed no differences in terms of mortality and ICU readmission rate.

Medical patients had a significantly higher ICU length of stay (8 ± 9 days versus 3.2 ± 4.5 in elective surgical patients and 6.2 ± 8.4 in urgent surgical patients, *p* < 0.001) (Table 4).

**Table 4:** Outcome and ICU LOS of ketonuric patients

	**Tot. *n* = 190**	**Medical *n* = 62**	**E surgery *n* = 60**	**U surgery *n* = 68**	***p*-value**
ICU readmission	*n* = 15 (8.5%)	*n* = 2 (3.9%)	*n* = 4 (7%)	*n* = 8 (14%)	0.15
ICU LOS days	6 ± 8	8 ± 9	3.2 ± 5.4	6.2 ± 8.4	< 0.001
ICU mortality	*n* = 16 (8.6%)	*n* = 7 (11.7%)	*n* = 1 (1.7%)	*n* = 8 (11.9%)	0.068

*E surgery* Elective surgery, *U surgery* Urgent surgery

## Discussion

There are no studies on the incidence of ketonuria and ketoacidosis in ICU patients. The intent of this study was to evaluate how much this clinical condition can lead to the development of a state of shock (or worsen a preexisting one), increase the risk of ICU readmission, and worsen ICU and hospital outcomes.

In our study population, patients that had undergone urgent surgery presented a higher rate of shock, higher levels of ketonuria, and lower albumin levels when entering ICU compared to medical patients. It has been widely studied how albumin levels alone in the early postoperative period do not exclusively indicate the nutritional status but rather the presence of an inflammatory state in response to surgical stress. Recent studies have shown a reduction of blood albumin levels by up to 43%, both during laparotomic and laparoscopic major abdominal surgery [32, 33]. A recent study by Komaromi et al. demonstrated how surgical stress leads to an even greater state of hypoalbuminemia compared to patients suffering from an acute inflammatory state who did not undergo surgery [34].

The production of ketone bodies in our study population is associated with fasting (> 12 h) in the absence of any carbohydrates supply; indeed, there were neither administration of non-nutritional calories. Fasting glycemia was lower in medical and urgent surgical patients compared to elective patients; anyway, mean glycemia levels were always > 120 mg/dL. This could be explained by the fact that both surgical stress and critical illness related to medical status (i.e., sepsis, trauma, pancreatitis, or respiratory failure) determine a metabolic dysregulation, activation of pituitary axis and hormone release, and activation of sympathetic system and stress response. These conditions determine an activation of a catabolic, stress-induced pathway, and dysregulation of the insulin secretion, thus leading to insulin resistance and ketone bodies production [2, 35].

In ICU patients, ketone bodies production, an adaptive response that can be useful to preserve organism in condition of stress and that induce autophagy that determine removal of several cellular damages, can be activated early [36] and is associated to the risk of promoting dysregulated ketoacidosis and SKA.

A large part of our surgical population underwent major surgery (pancreatectomy, esophagectomy, rectal resection, hepatectomy, splenectomy, pulmonary lobectomy, etc.) for cancer eradication, suggesting an even more complex state of catabolism and anabolic resistance linked to surgical stress and to the oncological status [37, 38].

There is a lack of study on ketosis in medical patients. It is known that sepsis can trigger an increase in ketone bodies through the same mechanism of insulin resistance in diabetic patients. The metabolic changes in sepsis can lead to a response like prolonged fasting, characterized by increased lipolysis and ketogenesis [39]. Additionally, circulating ketone body concentrations are also elevated in heart failure patients, in direct proportion to cardiac filling pressure.

Mostly in the surgical populations, the production of ketone bodies could be related to the presence and persistence of a harmful hypercatabolic state. Another focal point in the perioperative period is the evaluation of the risk of malnutrition. The two mostly used scores are the Nutritional Screening 2002 (NRS 2002) [40] and the NUTrition Risk in the Critically ill (NUTRIC score); the latter also considers IL-6 levels and therefore the inflammatory state [41]. Modified NUTRIC has been used to assess the risk of malnutrition in ICU patients, and a value greater than 5 has been related to high risk of malnutrition and adverse clinical outcome [42]. In the study population, mean mNUTRIC score was lower than the threshold for patients at high risk of malnutrition. However, we excluded from the analysis several patients with high SOFA score and APACHE II score to evaluate the impact of ketonuria in a selected subgroup of patients without several harmful clinical condition (i.e., septic shock, AKI, intoxication) and without chronical condition that are strongly related to malnutrition (CKD, COPD, diabetes). In this population, mNUTRIC could not be the best score to assess patients at high nutritional risk. NRS 2002 or MUST score was not used due to lack of information regarding preoperative conditions and weight loss.

In our analysis, all patients fasted more than 12 h. Ketonuric patients with shock have a significantly lower BMI than patients without hemodynamic instability. These data are in line with several studies that showed high mortality risk after surgery in patients with low BMI [43–46].

In our study, the lack of difference in terms of mortality and ICU length of stay between elective and urgent surgical ketonuric patients could be related to the older age and higher CCI of elective patients (but with lower SAPS II score), more exposed to catabolic injury and risk of perioperative malnutrition. Another explanation could be that all patients were treated early, within 24–48 h, with oral feeding, enteral nutrition through nasogastric or nasojejunal tube, or with glucose infusion. Although the heterogeneity in the clinical treatments did not allow a categorization of patients based on the treatment received, early nutrition and screening for ketonuria carried out in all patients at ICU admission could reduce protein wasting and the catabolism. Sawada et al. have shown how the use of glucose even in the intraoperative phase is able to inhibit lipolysis and proteolysis in patients undergoing surgical operations lasting more than 6 h [47]. Other biochemical exams should be used to detect catabolism, as serum urea/creatinine ratio that together with ketone bodies dosage could be a useful tool to properly detect the metabolic status of the patients [48].

This study shows how pre-, peri-, and postoperative nutritional optimization must be focused on reducing the catabolic effect of surgical stress in favor of optimizing anabolism and reducing anabolic resistance.

In conclusion, in ICU patients, multiple factors can increase ketone bodies. The turnover of ketone bodies involves interorgan and intercellular shuttles governed by integrated complex regulatory mechanisms. They also interact with nuclear ribonucleoproteins, inhibit histone deacetylases (HDACs), modify histones and other proteins posttranslationally, and influence oxidative stress. Anyway, the role of ketone bodies and their mechanisms of action are still under investigation [49]. Our hypothesis is that hemodynamic instability in ketonuric patients is multifactorial and could also be considered an expression of metabolic instability associated with an hypercatabolic state in conditions of malnutrition. We believe that ketonuria and hemodynamic instability are alert signals indicative of a more severe clinical state that require tempestive evaluation and subsequent treatment.

Nutrition in ICU is still a field of debate, and there are no biomarkers that are sensitive and specific for the evaluation of anabolic or catabolic states. In our opinion, the dosage of ketonemia or ketonuria, together with other clinical and laboratory findings, could be useful to better evaluate the metabolic status of patients [50].

Our study has some limitations: it is a retrospective observational study on a ketonuric population without a control group; ketone levels were measured in urine and not in blood; in surgical patients, ketones and albumin levels were measured only in the postoperative period, and we have no data regarding the preoperative levels; and the therapeutic approach was not standardized and was based on the choice of the clinician.

## Conclusions

This retrospective observational descriptive study showed how patients with ketonuria, hypoalbuminemia, and low BMI at ICU admission have high risk of hemodynamic instability and shock. Ketonuric surgical patients compared to medical patients are exposed to more severe catabolic trigger and are at high risk of malnutrition and anabolic resistance. In critically ill patients, early recognition and treatment of starvation ketoacidosis could reduce the incidence metabolic dysregulation, hemodynamic instability, and shock. The consequent downregulation of the catabolic pathway could reduce anabolic resistance and stimulate anabolic pathways. Further, prospective studies are needed to confirm these results.

## Data Availability

The dataset can be requested to simona.tantillo@ausl.bologna.it or irene.ottaviani@ausl.bologna.it.

## Abbreviations

AKA: Alcoholic ketoacidosis
AKI: Acute kidney injury
APACHE: Acute Physiologic Assessment and Chronic Health Evaluation
BMI: Body mass index
CCI: Charlson Comorbidity Index
COPD: Chronic obstructive pulmonary disease
DKA: Diabetic ketoacidosis
ICU: Intensive care unit
KDIGO: Kidney Disease: Improving Global Outcomes
mNUTRIC: Modified NUTrition Risk in the Critically ill
MUST: Malnutrition Universal Screening Score
NRS 2002: Nutrition Risk Screening 2002
NUTRIC: Nutrition risk in critically ill
SAPS II: Simplified Acute Physiology Score II
SKA: Starvation ketoacidosis
SOFA: Sequential Organ Failure Assessment

## Data availability

No datasets were generated or analysed during the current study.
